# The Protein Information Management System (PiMS): a generic tool for any structural biology research laboratory

**DOI:** 10.1107/S0907444911007943

**Published:** 2011-03-18

**Authors:** Chris Morris, Anne Pajon, Susanne L. Griffiths, Ed Daniel, Marc Savitsky, Bill Lin, Jonathan M. Diprose, Alan Wilter da Silva, Katya Pilicheva, Peter Troshin, Johannes van Niekerk, Neil Isaacs, James Naismith, Colin Nave, Richard Blake, Keith S. Wilson, David I. Stuart, Kim Henrick, Robert M. Esnouf

**Affiliations:** a CSED, STFC Daresbury Laboratory, Warrington WA4 4AD, England; b EMBL–EBI, Wellcome Trust Genome Campus, Hinxton CB10 1SD, England; c York Structural Biology Laboratory, Department of Chemistry, University of York, Heslington, York YO10 5DD, England; d Division of Structural Biology, Wellcome Trust Centre for Human Genetics, University of Oxford, Roosevelt Drive, Oxford OX3 7BN, England; e School of Life Sciences, University of Dundee, Dundee DD1 5EH, Scotland; f Department of Chemistry, University of Glasgow, Glasgow G12 8QQ, Scotland; g Biomolecular Sciences Building, University of St Andrews, St Andrews, Fife KY16 9ST, Scotland

**Keywords:** laboratory information-management systems, protein production, Java web applications, data management and databases

## Abstract

The techniques used in protein production and structural biology have been developing rapidly, but techniques for recording the laboratory information produced have not kept pace. One approach is the development of laboratory information-management systems (LIMS), which typically use a relational database schema to model and store results from a laboratory workflow. The underlying philosophy and implementation of the Protein Information Management System (PiMS), a LIMS development specifically targeted at the flexible and unpredictable workflows of protein-production research laboratories of all scales, is described. PiMS is a web-based Java application that uses either Postgres or Oracle as the underlying relational database-management system. PiMS is available under a free licence to all academic laboratories either for local installation or for use as a managed service.

## Abstract 2

The Protein Information Management System (PiMS) is described together with a discussion of how its features make it well suited to laboratories of all sizes.

## 1. Introduction

Recent decades have seen rapid advances in the techniques of protein production, crystal growth and structure determination. These methodological advances have been accelerated by structural genomics initiatives, which have aimed to develop generic techniques that can be parallelized and optimized. Despite the increasing numbers of experiments performed, the primary medium for recording the results of these experiments remains the laboratory notebook. Features of the notebook which make it popular include (i) the free-format nature of the information recorded, (ii) the ability to keep the notebook at the bench, (iii) the ability to attach ‘images’ such as gels and chromatograms and (iv) the personal nature of each notebook. This latter feature is both a strength and a weakness: someone working alone can optimize their personal data-recording method, but it may be nearly impossible for others to decipher the information later. Thus, laboratory notebooks may be very convenient in the short term or for one person, but in the long term key information can often be lost.

The potential benefits of electronically recording laboratory information have long been recognized (Robinson, 1983 ▶; McDowall, 1988 ▶), especially for large-scale projects such as the Human Genome Project (Hunkapiller & Hood, 1991 ▶) and subsequent structural genomics projects (both commercial and academic; for examples, see Peat *et al.*, 2002 ▶; Goh *et al.*, 2003 ▶; Zolnai *et al.*, 2003 ▶). Electronic data can be shared over the internet, enabling close collaboration between remote researchers. Defining standards means that the recorded data are meaningful to everyone. Several structural biology standards have been defined either for reporting progress or for depositing data. Examples of the former include TargetDB (Chen *et al.*, 2004 ▶) and PepcDB (http://pepcdb.pdb.org/) and examples of the latter include the Protein Production Data Model (PPDM; Pajon *et al.*, 2005 ▶), the SG Knowledgebase (Berman *et al.*, 2009 ▶) and, of course, the wwPDB itself (Berman *et al.*, 2003 ▶). Electronic recording of data is also beneficial, if not essential, when working with miniaturized automated experiments in plate formats and where either a researcher works on many projects in parallel or where many researchers work on a single project. There are additional benefits from the management point of view in providing an overview of progress through to costing and scheduling research.

The popularity of electronic data recording remains low, however, as systems are often seen as slow, difficult to manage, inconvenient to use or even counter-intuitive. Moreover, it is not usually convenient to have a computer at hand on the bench-top, so data recording is either a (wasteful) two-stage process going *via* paper or prone to be incomplete. There are many approaches to recording data electronically. One extreme model is to allow the recording of completely unstructured data: essentially the same as using a shared MS Word document and inserting images into it as necessary. This approach is the closest to current laboratory work practice and is usually termed an electronic laboratory notebook (ELN). Its main drawbacks are related to the lack of standards. Taylor (2006 ▶) and Wright (2009 ▶) provide reviews of the current state of ELN software. An intermediate level of structure is to populate predefined spreadsheets such as MS Excel. This is well suited to parallel experiments, where some parameters are common to a set of experiments, where the data which should/can be recorded are predefined or where it is simply recording progress along a linear workflow. However, spreadsheets rapidly become unwieldy and difficult to use as the processes become more complicated.

Managing the complexity and richness of research workflows requires full-blown laboratory information-management systems (LIMS), which are typically underpinned by relational databases. LIMS usually have a model for the laboratory workflows that they manage and they encapsulate this knowledge in the database schema. LIMS can be very effective when single processes are performed many times, such as in testing and quality-assurance (QA) scenarios, where the process is well understood in advance and unlikely to change or where the experiment is inherently high-throughput, such as proteomics (reviewed in Stephan *et al.*, 2010 ▶). Such LIMS can define, and even demand, that specific information is entered and can allow processes to be described in a manner that satisfies stringent regulatory demands, such as for clinical data.

Work in research laboratories does not usually fit well with rigidly defined data models. A key skill of the researcher is that they can respond to unexpected outcomes and create new experiments on the fly. Where the data model is reflected directly in the structure of database tables it is very difficult for a LIMS to be adaptable without extensive reprogramming. Therefore, such LIMS will always lag significantly behind laboratory practice and hence data cannot be recorded as they are produced. An alternative approach to LIMS development is to create a data model built around abstract concepts, whereby the modelling of any particular laboratory process is then a reconfiguration, rather than a reprogramming, of the application. This method has three potential drawbacks. Firstly, it usually requires a well trained and skilled LIMS ‘power user’ who can design and perform the necessary reconfigurations. Secondly, the user interface tends to be based on the abstract components of the underlying data model, making it un­intuitive and unwieldy to use. Thirdly, operations that seem simple from the user’s perspective may map to complex database manipulations on heavily used tables, giving unacceptably poor performance. Thus, the development of LIMS for research remains a significant informatics challenge, as shown by their relative scarcity in academic science laboratories. Examples related to structural biology include LISA (Haebel *et al.*, 2001 ▶), XTRACK (Harris & Jones, 2002 ▶), SESAME (Zolnai *et al.*, 2003 ▶), CLIMS (Fulton *et al.*, 2004 ▶), HALX (Prilusky *et al.*, 2005 ▶) and MOLE (Morris *et al.*, 2005 ▶).

This paper describes the underlying philosophy and implementation of the Protein Information Management System (PiMS), a LIMS development specifically targeted at the flexible and unpredictable workflows of protein-production laboratories of all scales. It uses a relatively simple generic data model, but considerable effort has been devoted to simplifying and optimizing the user interface and configurability of the system. PiMS is a web-based Java application underpinned by a relational database management system. PiMS was primarily developed as part of the BBSRC SPoRT initiative to support the work of the Membrane Protein Structure Initiative (MPSI), the Scottish Structural Proteomics Facility (SSPF) and the Oxford Protein Production Facility (OPPF), with additional contributions from the CCP4 project and the MRC. PiMS is available to all academic laboratories free of charge under a licence similar to that of the CCP4 suite. PiMS can either be installed locally or else data can be stored using the managed PiMS service.

## 2. Methods

### 2.1. Underlying data model

**Figure 1:**
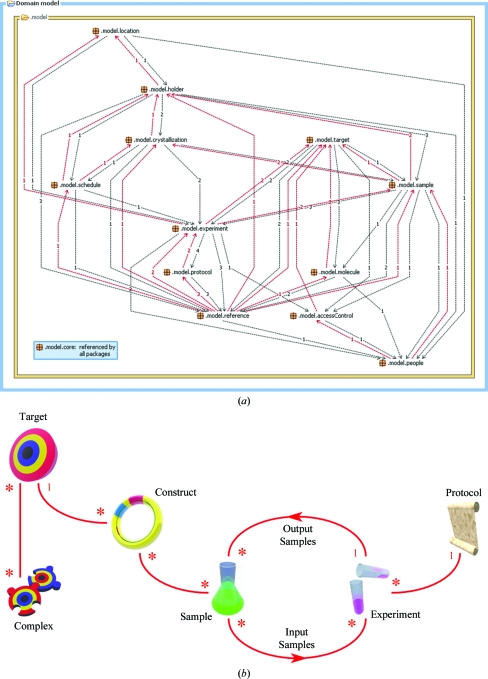
An overview of the PiMS data model. (*a*) Diagram showing the 13 key data-model packages (and the number and direction of the relationships between them) that describe all aspects of tracking experimental work in a collaborative multi-user environment. The package .model.core is referenced by all other packages and defines properties which can be recorded for any object in the database. (*b*) A simplified diagram showing the relationships between the essential PiMS concepts. The icons are used throughout PiMS to indicate object types. The ‘1’s and ‘*’s on the red lines indicate one-to-many and many-to-many relationships. For example, a Sample is the output of a single experiment, but it can be used as the input to many experiments.

PiMS has a sophisticated and complex data model developed from the PPDM (Pajon *et al.*, 2005 ▶). This model has been divided into 13 key packages, many of which relate to abstract concepts (Fig. 1 ▶ *a*), but the use of PiMS does not require any understanding of this underlying data model. The PiMS data model was originally defined using the ObjectDomain UML modelling tool. Since PiMS version 2.0 the use of this tool (and the source files it autogenerates) has been discontinued and modelling work is now performed directly using Hibernate tools, which can then generate UML diagrams. The full PiMS data model is available from the project website (http://www.pims-lims.org/auto/java/javadoc/).

While knowledge of the data model is not required, it is very helpful to have a basic grasp of the essential PiMS concepts, which are summarized in Fig. 1 ▶(*b*) and discussed below. In short, Experiments (based on Protocols) are performed on Input Samples to produce Output Samples, which can in turn be used in further experiments *etc*. Each PiMS record is part of a Lab Notebook, which is private to a user or group of users, providing a mechanism for keeping data from different projects separate. A laboratory can create as many Lab Notebooks as necessary to segregate the data and provide access control. Most items in the normal workflow (including Protocols, Experiments, Targets and Samples) have the properties of a Lab Notebook Page. This property allows items to be associated with names, people, access permissions, attached files and images, external database references *etc*.

### 2.2. Standard and user-defined protocols

PiMS avoids the use of tightly predefined workflows. This provides flexibility and makes it easier for the system to record *ad hoc* experimental information and other unexpected data items. Instead, PiMS is built around the idea of Protocols, which are user-definable reusable experiment templates. On installation, PiMS comes with a set of (presently) 26 default Protocols (Table 1 ▶) which are appropriate for most protein-production work or which can be customized further. A Protocol defines the Sample types (both Input Samples and Output Samples) that relate to an experiment as well as specifying which parameters/results should (or must) be defined. A Protocol can also specify default values for selected parameters. As well as being given a name, Input Samples and Output Samples are assigned a type. Sample types are important, since the Output Sample type of one experiment must match the Input Sample type of the next experiment to be performed. Through the use of types, PiMS is able to give the user sensible options and shorter lists of Samples from which to choose. For example, there is no point in allowing a PCR product to be the input for a protein-purification experiment.

**Figure 2:**
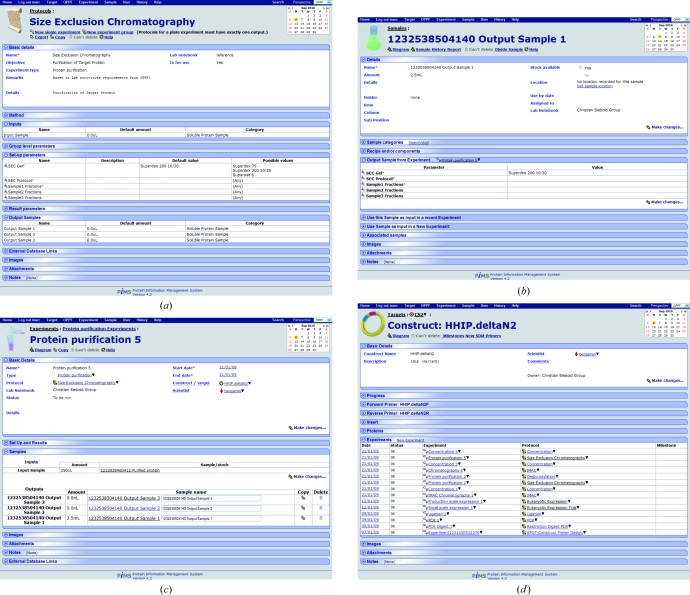
Screenshots from standardized PiMS pages for common entities. The relevant data are presented in blocks, which can be expanded as desired to see the full details. (*a*) A Protocol page showing expanded data blocks for inputs, setup parameters and output samples. (*b*) A Sample page showing an expanded data block for the setup parameters of the Experiment that produced the Sample. (*c*) An Experiment page for the Experiment based on the Protocol in (*a*) that produced the Sample in (*b*). The samples data block is expanded. (*d*) A Construct page for the Sample shown in (*b*). The data block showing the list of recorded experiments based on that Construct is expanded.

An example Protocol is shown in Fig. 2 ▶(*a*). The screen is divided into several key sections: (i) the basic details (header) section, which includes the Protocol name and type; (ii) the methods description, which forms part of a Protocol reference library; (iii) the description of Input Samples; (iv) the setup parameters section, which describes the setup of an experiment; (v) the result parameters section, which describes the progress of an experiment; and (vi) the description of Output Samples. The sections at the bottom of the figure are common to many PiMS items and allow external database links, images, files and other notes to be attached to items.

An actual Experiment is an instance of a Protocol. Protocols can be created or (more usually) modified from existing ones. However, to preserve the integrity of the PiMS database normal users are not allowed to modify or delete Protocols which have already been used. If the Protocol is in use, such as in the example shown in Fig. 2 ▶(*a*), there is a button just below the Protocol name for creating a copy of the Protocol with a different name, which can then be edited. The standard set of Protocols is visible to all users, while customized Protocols are stored in your personal Lab Notebook but can be shared with other users of the system or exported to other PiMS installations for use by other laboratories.

### 2.3. Standard user-interface features

Nowadays, time spent learning an application is considered wasted. Instead, the drive is to create ‘intuitive graphical user interfaces’. In practice, this means developing interfaces that conform to a set of standards widely used in software development. When the project started, the PiMS developers faced a dilemma between developing a web application and accepting the limitations of such interfaces or developing a Java application which had to be installed (and supported) on all client machines but which allowed many more usability features to be exploited. The decision to develop a web application, while removing a large support and compatibility issue, gave the user-interface developers a serious challenge and much of the PiMS development effort has gone into refining, standardizing and optimizing a web-based interface to deliver many of the features associated with locally installed applications. Fig. 2 ▶ shows examples of commonly used standardized PiMS pages. User-interface guidelines for PiMS are available on the project website (http://www.pims-lims.org/).

### 2.4. Technical details

PiMS has been developed as a Java-based web application to work with Java 1.5 or later and makes extensive use of AJAX technologies. PiMS also makes use of the BioJava, dot and batik packages. Extensive JUnit testing is performed for all software builds. PiMS requires an underlying relational database management system (RDBMS), which can be either Oracle (Oracle Corporation, Redwood Shores, California, USA) or Postgres (http://www.postgresql.org/). The mapping between the object-oriented application and the relational database is handled by Hibernate. Conversion to work with other RDBMSs would be feasible for a competent database programmer. PiMS requires no software installation on the client machine, which can be Windows, Linux or Macintosh, and is supported for Internet Explorer 7, Mozilla Firefox 2 and Safari 4 (and later versions).

### 2.5. Installation of PiMS and the PiMS service

Upon completion of the PiMS licence agreement, PiMS can be downloaded from http://www.pims-lims.org/. Commercial organizations are required to contact the PiMS team directly for licensing. Typically, only modest server hardware is required to install PiMS. The PiMS service and OPPF servers both perform well with 4 GB memory, of which Tomcat uses 1 GB, but with current multicore servers 16 GB memory would be preferred. The server can either run Windows or Linux and needs to have the Tomcat 6 application server. The database can reside either on the same machine or on another system. The minimum requirement is either Postgres 8 or Oracle 10. If PiMS is to be installed locally, then it is the responsibility of the laboratory to implement suitable disaster-recovery procedures and to control access to the system. The overall installation process, consisting of a series of standard package installs, is relatively straightforward for a competent computer user with some system-administration experience.

Since many smaller laboratories have little IT support, a centralized public PiMS service has been implemented that uses hardware provided as part of the preparatory phase of the EU INSTRUCT project and managers of the PiMS service keep the software updated and make regular backups. It also uses the National Grid Service (NGS) Oracle installation. To use the PiMS service, users simply need to register on the website (http://pims.instruct-fp7.eu/) and start recording data. Data access is secure and strict access controls maintain data segregation. There is no need for any local software installation or management. Data held in one PiMS installation can be exported in bulk to another installation and work is in progress to allow a selection of specific records to be exported.

For further information on PiMS, online tutorials, help pages, licensing information, access to the PiMS server or to contact the development team, please visit the PiMS website at http://www.pims-lims.org/.

## 3. Discussion

### 3.1. Key PiMS concepts

**Figure 3:**
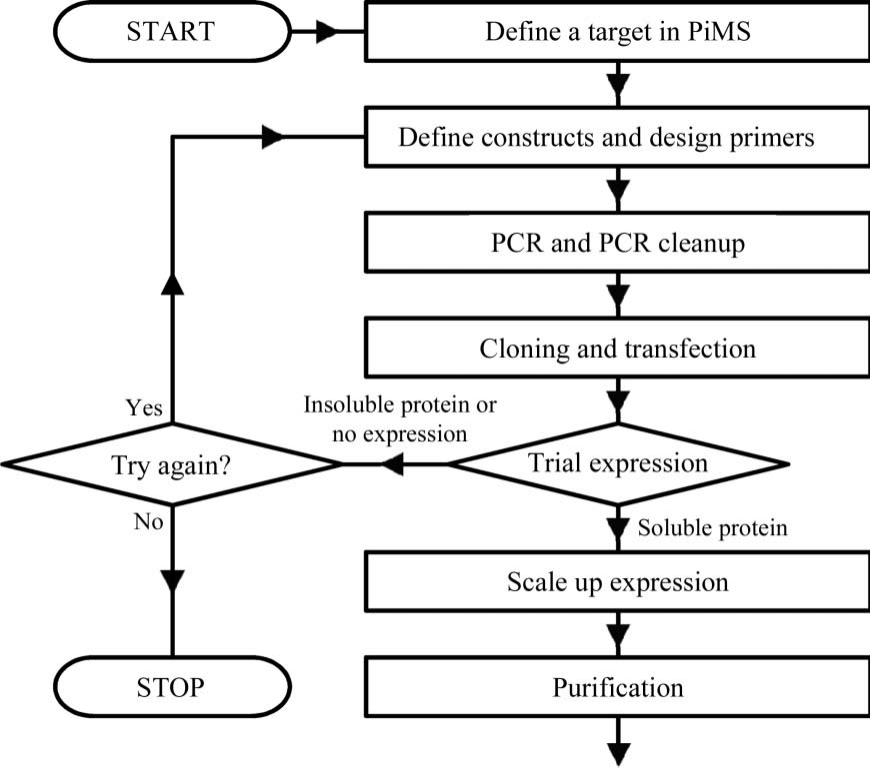
A schematic showing the series of experimental steps that might be involved in producing a sample of purified protein. Apart from target definition, all the stages shown correspond to one or more PiMS Experiments. Each Experiment (apart from construct definition/primer design) uses one or more Input Samples and (apart from trial expression, where only knowledge of expressibility is required) produces one or more Output Samples.

A basic grasp of the key PiMS concepts (Fig. 1 ▶ *b*) makes learning the system much more straightforward. To emphasize the simplicity and usability of the system, the discussion below focuses on the most common use case: the application of PiMS to record progress toward the production of pure protein samples. We will consider this process from the start (an example workflow is show in Fig. 3 ▶), although PiMS can be used to record data starting at any stage in the process (*e.g.* from transfected cells).

#### 3.1.1. Targets

The first stage is to declare the protein target that you are working on and PiMS has several options (discussed below) for easing this process. A PiMS Target describes a full-length protein (the translation of a full-length open reading frame) and its associated DNA sequence. It serves as a place to link in references to external sources of information.

#### 3.1.2. Constructs

PiMS Targets do not relate to physical samples. The missing link is provided by the PiMS Construct, the entity that is used to declare which physical samples are intended to be worked on and the relevant protein sequences. PiMS provides tools for construct design, although externally designed constructs can be entered/uploaded (using extensions written for the OPPF). This stage is performed by the virtual ‘construct-design’ PiMS Experiment (see below). The Output Samples (see below) created by the construct-design experiment include all the PiMS Samples required for the first experimental step (usually PCR).

#### 3.1.3. Samples and experiments

At the very heart of PiMS are the two interdependent concepts of Samples and Experiments. A Sample is the definition of a physical sample: as expected, it can have a creation date, label, location, owner and a description of what it contains. The purpose of samples is to be used as Input Samples for Experiments, which in turn may produce Output Samples. The latter samples can then be fed into further experiments, thereby building up complete workflows. The link between a Sample and an Experiment also enables the building of custom functionality, such as the sequencing sample tracking described in more detail elsewhere (Troshin *et al.*, 2011 ▶).

#### 3.1.4. Protocols

A PiMS Experiment is an instance of a PiMS Protocol: a reusable experiment template that stores the information about how experiments of that type should be performed. A key feature of Protocols is that they can be modified to create new Protocols, thereby allowing a laboratory to record experiments in whatever manner (*e.g.* level of detail) it considers appropriate. Extensive use of Protocols allows PiMS to avoid the need for workflows (pre-definitions of the laboratory practice), something that makes most other LIMS developments too inflexible for research environments. As stated earlier, PiMS comes with a set of default protocols which can be used ‘as is’ or customized to meet local requirements.

#### 3.1.5. Sample typing

**Figure 4:**
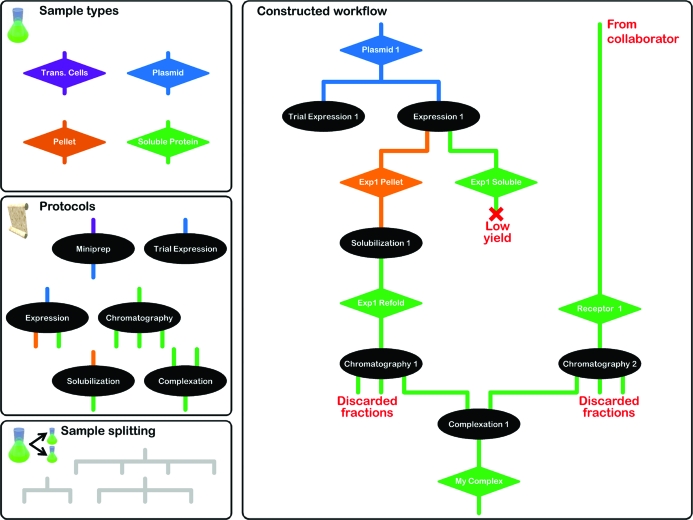
A schematic showing how Sample types are used in conjunction with Protocols to build up a PiMS workflow. The left-hand boxes show the Sample type and Protocol definitions used in this example. The right-hand box shows the constructed workflow where each Sample now has a name (colour-coded by Sample type) and each Experiment has a name based on the Protocol used. Samples are implicitly split by choosing them for multiple Experiments.

Most items in PiMS are typed in one way or another. This is normally performed automatically and allows PiMS to offer the user sensible choices most of the time. Samples, for example, may be DNA, cells, soluble protein *etc.* By including in a Protocol definition the types of Sample which can be used as inputs to and produced as outputs of an Experiment, PiMS is able to make sensible suggestions. For example, PiMS will not suggest plasmids as input samples for nickel-affinity chromatography experiments. Conversely, the declaration of the input/output sample types for Protocols means that PiMS will not suggest that protein samples are used for PCR experiments (shown schematically in Fig. 4 ▶). This typing extends further; for example, Protocols are grouped together by type (*e.g.* protein purification). This enables searches to be performed over all Protocols of a specified type, not just over experiments based on a single Protocol.

#### 3.1.6. Complexes

PiMS Complexes are intended to represent biological complexes, rather than artificial com­plexes created in a laboratory. Thus, Complexes are declared to be created from two (or more) PiMS Targets. Typically, many constructs for target proteins will be tried before a suitable model experimental system is discovered. To simplify working with complexes in PiMS, the software automatically checks whether any Sample that is linked (usually *via* Constructs) to multiple Targets could relate to a declared Complex. PiMS considers work on any such Sample to be part of work toward the biological Complex. Furthermore, PiMS attempts to detect where complexes might have been formed; for example, the outputs of complexation or co-expression experiments. Working with PiMS Complexes will be described in more detail elsewhere (Savitsky *et al.*, 2011 ▶).

### 3.2. Getting started: creating targets and constructs

Prior to using PiMS, a user will need to have access to a PiMS system and have a valid username/password combination. Researchers wanting to use the PiMS service should follow the ‘Request PiMS UserID’ link on the PiMS service homepage (http://pims.instruct-fp7.eu/). If PiMS is being installed locally, then creating these User accounts is currently part of the responsibility of the person installing the system. Note that PiMS uses Tomcat’s authentication facilities to answer the question ‘Who are you?’, but has its own access-control facilities to decide ‘Are you allowed to see these data?’ As well as the new User, the administrator may also need to create a personal Lab Notebook (unless all work will be recorded in a pre-existing project Lab Notebook) and to create/modify User Groups (which provide the access-control mappings between Users and Lab Notebooks).

**Figure 5:**
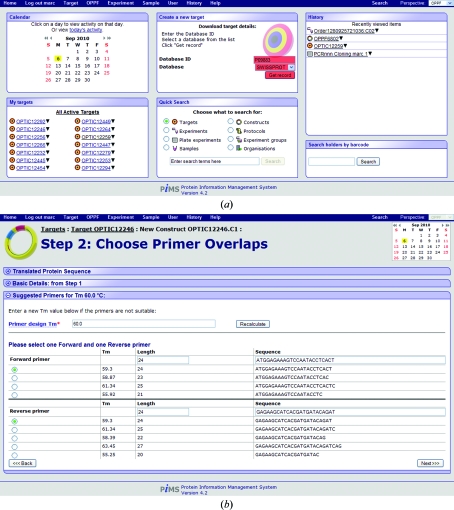
Screenshots showing simple use of PiMS. (*a*) The user’s PiMS home page. Blocks on the page show the most recent activity and common actions by that user. The boxes highlighted in red show the simplest set of actions required to define a Target in PiMS (based on an external sequence database). (*b*) The output of the primer-design code, part of the standard Construct design process.

Once successfully logged on to PiMS, the user is presented with the home page (Fig. 5 ▶ *a*) and can begin to store data. The typical starting point is the declaration of relevant Targets against which work will be recorded. As well as providing the link to Constructs and Samples and being stores for external bioinformatics information, Targets are assigned to Lab Notebooks to determine the (default) access control for associated experimental data. The declaration of all possible information for a Target can be quite laborious, so tools are provided to ease the process. The simplest tool is located on the user’s PiMS home page (Fig. 5 ▶ *a*), which merely requires the user to enter a database accession number from which PiMS uploads the database entry and parses out the relevant data. Although DNA and protein sequences are not both required to declare Targets (indeed, Targets can represent noncoding regions of DNA), supplying a full DNA sequence is required to make full use of the PiMS construct-design facilities. A Target is normally intended to represent a biologically relevant entity, fitting naturally with the definition of PiMS Complexes as real biological assemblies.

Having declared a Target, the user can now design Constructs. PiMS has built-in code for primer design which is simple to use and caters for most needs. Firstly, the user defines the starting and stopping amino acids, whether an N-­terminal methionine is required and what tags are required. PiMS then suggests primers, based on a user-selectable target melting temperature (Fig. 5 ▶ *b*). This Construct definition and primer-design process is a special hybrid Experiment that requires no input but produces all the output Samples necessary to move on to a PCR experiment: the template and the forward and reverse primers. Two PiMS installations (the OPPF and MPSI) have incorporated support for pre-existing construct-design tools and in these cases local customization provides import functions for constructs designed with *OPINE* (at the OPPF; Albeck *et al.*, 2006 ▶) or using *VectorNTI* (at the MPSI; Troshin *et al.*, 2008 ▶).

In practice, primers are often ordered in batches from external companies and it is especially important that the primers are properly matched to the template. By creating a layout of Constructs in plate format, PiMS will create corresponding template and primer plates in the correct format. One feature of constructs is that they store the expressed, final and any other relevant protein sequences. Coupled with the fact that all protein samples are considered to be derived from a parent construct, this means that constructs provide a single reference point for determining the actual sequence of a protein in a sample at any stage in the workflow. Sequences stored in PiMS can easily be aligned with each other and with external sources of protein sequence, such as the PDB or TargetDB, to show close matches. Furthermore, there is a simple link provided to upload a PiMS sequence to the *TarO* bioinformatics analysis pipeline (Overton *et al.*, 2008 ▶).

### 3.3. Creating workflows: samples and experiments

The central relationship in PiMS is the link between Samples and the Experiments that consume or produce them (Fig. 1 ▶ *b*). The user can interact with this circular relationship either from the point of view of the Sample or the Experiment. PiMS has a series of standardized pages for viewing common PiMS objects (Fig. 2 ▶). From the Sample view page, the user is given the option to use the Sample as (part of) the input to a new Experiment derived from an existing Protocol. The choice of Protocol that is offered is filtered by requiring that the type of one of the Input Samples matches the type of the current Sample. When the Protocol is selected, the user is given the chance to complete the other details and then to create an instance of the Protocol (an actual PiMS Experiment). Conversely, the user can decide which Protocol they want to use for their next Experiment, select it and then provide all the details of the Samples that are to be used and the parameters that need setting.

One problem with recording laboratory data electronically is exactly how and when data are recorded. Just as with laboratory notebooks, data can be entered before the experiment starts as part of the planning, during the course of an experiment (including recording unplanned extra notes) and after completion of an experiment when the final results are recorded. Some results (*e.g.* outsourced quality-assurance experiments such as sequencing) may only become available some time after an experiment completes. Most of the time, recording an *ad hoc* note in a LIMS is harder than in a laboratory notebook since the correct ‘page’ has to be located first. Furthermore, there is a need to log on to the system and it may be inconvenient (or impossible) to get access to a computer/keyboard from the experimental bench. Thus, in practice, data are usually recorded after experiments have been completed. The weakness of this model of working is that results end up being written on paper before entry into the system, leading to duplication of effort. If this double step could be avoided, perhaps by developing customized interfaces for portable touch-sensitive screen data-entry systems (*e.g.* iPad) that can be used with gloved hands, then PiMS would become easier to use than paper.

One characteristic feature of PiMS is that it avoids the need to predefine workflows by making extensive use of user-definable Protocols which form templates for actual Experiments. Nevertheless, by declaring a set of Samples and Experiments, PiMS can build up the actual workflow and present this graphically and interactively to the user (see below). One drawback of this approach is that since each workflow is built up on the fly, it can be difficult to compare results between different workflows.

### 3.4. Navigation in PiMS

The key to developing a successful LIMS is its usability. There are several aspects to this: ease and efficiency of data input, having a standardized and predictable user interface, having efficient and intuitive navigation through the data and an ability to move easily around the system. The discussion above has highlighted aspects other than navigation. The basic navigation system in PiMS is by searching for the required entity (a Sample, Experiment *etc*.) and then clicking on buttons to create new PiMS entities. Since the number of entities in a PiMS database is large, search features have been added to make this more efficient. Furthermore, users tend to reuse/revisit the same entity repeatedly and having viewed one entity makes it likely that the same entity will be revisited soon. PiMS exploits this in two ways: (i) drop-down lists ‘remember’ what has been accessed recently and present these choices first and (ii) the home page shows the most recently used entities, providing a one-click route to accessing them.

**Figure 6:**
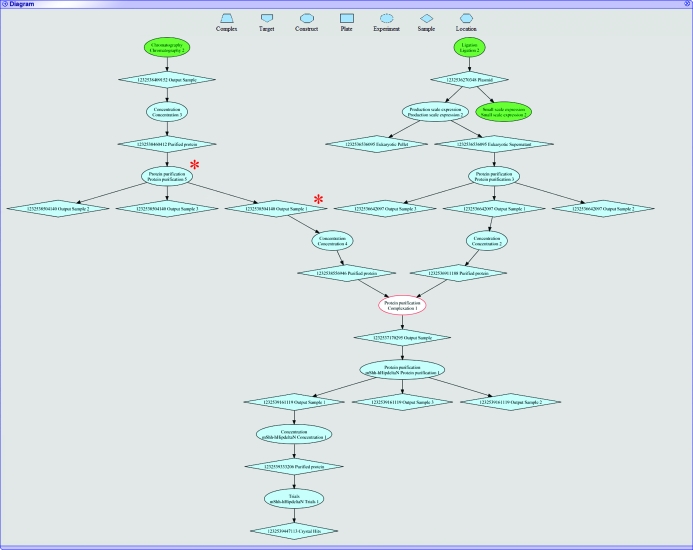
An example of an interactive workflow diagram automatically generated within PiMS. Ellipses represent experiments and diamonds represent samples. While most components are coloured blue, the green shapes show where the diagram has been truncated to avoid excessive complexity on the screen and the white shape with the red border indicates the starting point for the current diagram. Clicking on a shape navigates to the standard PiMS page for that object. For example, clicking the shapes indicated by red asterisks navigates to either the experiment page or the sample page in Figs. 2 ▶(*c*) and 2 ▶(*b*), respectively.

A second navigation system has been implemented based on intuitive and interactive diagrams of workflows (Fig. 6 ▶) which are constructed on the fly from data recorded in PiMS. In these diagrams, each major PiMS entity is represented by a different shape and arrows show how entities feed into each other (*e.g.* how Samples feed into Experiments, which in turn produce more Samples). Entities are usually coloured blue, except for the one on which the diagram is based (which is white with a red border). Diagrams can become complex and so they are truncated to show the only the most closely linked entities. These points of truncation are coloured green, showing that there is further information. The value of these diagrams is that they are interactive: clicking on one entity goes to the page that describes it in detail. Returning to the diagram view from that entity page re-centres the diagram on that entity, perhaps revealing new information. In this way it is possible to scan quickly over an entire workflow, however complex it may be. The diagram system has proved to be popular and indeed has become the main navigation route for some users. Further development is likely, with small diagrams having enhanced functionality appearing on all pages, *e.g.* the ability to perform common tasks (such as recording new experiments) by right-clicking on the object of interest.

Whichever method of navigation is used, PiMS will check that the user is only presented with information that they are entitled to read (or modify). Most pages (or diagram blocks) correspond to separate Lab Notebook Pages and therefore their access control can be independently modified. However, in real-use cases Targets are assigned to Lab Notebooks and this is used to determine automatic access control for all other entities relating to that Target.

### 3.5. Browsing data in PiMS

Since the purpose of recording information is to recover it later, the ease and convenience of reporting, searching and comparing information held in a PiMS database is crucial. PiMS has features covering all three forms of analysis. Firstly, a complete Sample History Report is available for any Sample by following the relevant link (directly below the Sample name on the Sample page; Fig. 2 ▶ *b*). This report (screen-based, as a PDF document or as SPINE2Complexes-compliant XML) details all the experimental stages that have led to the production of the Sample. The workflow diagrams (above and Fig. 6 ▶) are also embedded in the PDF reports. Second, PiMS has search facilities to aid in locating data. A simple interface to search for text associated with a particular type of object is present on the user’s PiMS home page (Fig. 5 ▶ *a*). Thirdly, PiMS data can be compared with each other. The results of these comparisons can be presented in tabular or (in some cases) graphical form. One common scenario is to track ‘cohorts’ of Samples through a series of plate-based experiments, where a graphical view can be generated to show which samples were successful in PCR, cloning or expression trials *etc*. Such flexible browsing methods are one of the most compelling reasons for switching to electronic data recording.

One report view that has not yet been fully implemented in PiMS is the calendar-based report, which is the closest equivalent to the traditional paper notebook. Work is in progress to provide this facility in the hope that the ability to print a nicely formatted comprehensive work record will encourage users to switch to electronic record-keeping in preference to paper notebooks.

### 3.6. Current usage of PiMS

The PiMS LIMS supported the work of the two main SPoRT consortia funded by the BBSRC: the SSPF and MPSI. Furthermore, PiMS has been the main LIMS used at the OPPF since 2007. All of these sites have a significant level of automation and the value of LIMS in systematically recording and managing the data produced by robotic systems is clear. In more ‘traditional’ laboratory settings PiMS is being used within the Division of Structural Biology (STRUBI), Oxford, at the York Structural Biology Laboratory (YBSL) and to support some of the non-SPoRT work of laboratories in Leeds, Glasgow and St Andrews. Outside the original development consortium, a further 18 completed PiMS licences have been received, with non-UK installations in Germany, Spain, Portugal, Finland, Austria, China and, recently, the USA. The OPPF installation is the most heavily used and demonstrates that PiMS can scale effectively to meet the requirements of most (if not all) structural biology laboratories. As of September 2010, the OPPF PiMS has records for one complex (complexes were not a part of the original OPPF remit), 557 targets, 2735 constructs, 27 066 samples, 2496 simple (nonplate) experiments, 291 plate-based experiments (representing an additional 27 936 individual experiments) and 118 protocols (these figures do not include crystallogenesis experiments). The hardware which powers the OPPF PiMS server (and which also serves several other web applications) is a dual 2.4 GHz Xeon server with 4 GB memory running Windows Server 2003.

The public PiMS service was initiated in March 2009 with funding from the preparatory phase of the EU INSTRUCT project. The web application runs on dedicated hardware with a disk pool for uploading image (and other) data, while the back-end database capability is provided by the National Grid Service. The goal is twofold: to offer a convenient test system for attracting new users and to allow users from all laboratories to benefit from a managed PiMS service without the need for local software installation or IT management. The service now has 78 registered users from 20 sites and is being used to store live data. Data stored with the PiMS service remain the property of the user and can be returned to the user if required *via* an XML format, *e.g.* if the laboratory decides to install PiMS locally or requires the data for storage in a different system. Furthermore, PiMS provides a con­venient mechanism to report how any sample was created, providing all the information from all the experimental steps involved in its production, including gels and other images.

## 4. Conclusions

The potential benefits of electronic recording of laboratory data are clear, yet previous systems have failed to make a significant impact in the structural biology community. This is particularly surprising given the computer literacy of structural biologists compared with many other experimental life scientists and suggests that the cause may lie in areas such as ease of use and workplace practice. An obvious issue is that the main benefits of electronic data recording – the improvement in long-term data management, access to data by others and project management – are not of immediate benefit to those entering the data. Another major issue is that no two researchers record data in their notebooks in the same way, a lack of standardization that can only be addressed by electronic systems at the expense of perceived usability.

For miniaturized, automated and parallel experiments electronic data recording is all but obligatory and electronic information management is readily adopted as it is essential for capturing the wealth of information that is generated. In these cases, laboratory equipment often comes with its own control application and it is essential that any LIMS has reliable automatic transfer of data to and from the control application to increase efficiency and to avoid duplication and error. A sister application, xtalPiMS, which maintains the look and feel of PiMS but is optimized for the management of high-throughput crystallization trials is an example of such a case and will be described elsewhere. As research methods evolve, it is essential that information-management systems evolve with them. PiMS partly achieves this goal thanks to the flexibility of its Protocols, but continuous software development is also needed to provide tightly integrated support for automated processes.

The PiMS LIMS project has sought to meet the informatics challenges of structural biology head on and to provide a universal, flexible and easy-to-use (and easy-to-understand) system that is freely available for use by all academic laboratories. Given the limited success of previous endeavours in this area, PiMS is truly a piece of research software in itself, albeit one developed to professional standards of software quality. It has achieved a level of interest and uptake that no other general LIMS has matched and by this measure it has been a successful research project. Indeed, the lessons learned by the PiMS team during the development process have themselves been studied (Segal & Morris, 2008 ▶; Morris & Segal, 2009 ▶).

PiMS has not (yet) been able to revolutionize the way that structural biologists work. Further improvements to PiMS, particularly with respect to navigation and reporting, may change this situation. As more automated and standardized experimental techniques become commonplace, using PiMS to record (or even plan) experiments may become easier than using a paper notebook. Nevertheless, some sort of revolution is required: research funders are now stressing the scientific value of archiving and sharing experimental results and to perform this effectively requires an infrastructure such as that provided by PiMS. There is a tradition of sharing structures through the PDB (now wwPDB); more recently, crystallo­genesis data have enabled the design of improved screens. If such benefits are to be obtained for the earlier stages of laboratory work, then some changes to working habits and culture are inevitable.

## Figures and Tables

**Table 1:** The set of 26 Protocols supplied as part of the standard PiMS installation The Protocols are divided into groups reflecting the different stages of the protein-production pipeline. These Protocols are available to all users of a PiMS system. Users modify these Protocols by making a local copy of them and then making changes.

Protocol name	Input samples	Output samples
Processing DNA		
PCR	Primers; template	PCR product
PCR cleanup	PCR product	PCR product
PCR product digest	PCR product	PCR product
Ligation	PCR product; linearized vector	Ligated plasmid
Bicistronic cloning	2× PCR product; vector	Recombinant plasmid
Bicistronic InFusion	2× PCR product; vector	Recombinant plasmid
Vector digest	Vector	Linearized vector
Clone verification	Template	PCR product
Cell growth and protein expression		
Transformation	Plasmid; competent cells	Transformed cells
Culture	Transformed cells; culture medium	Transformed cells
Miniprep	Transformed cells	Purified plasmid
Trial expression	Plasmid	Protein
Large-scale expression	Plasmid	Pellet; supernatant
Solubilization	Pellet	Soluble protein
Processing protein samples		
Tag cleavage	Soluble protein; enzyme	Soluble protein
Chromatography	Soluble protein	Soluble protein
Size-exclusion chromatography	Soluble protein	Soluble protein
Complexation	2× soluble protein	Soluble protein
Concentration	Soluble protein	Soluble protein
Protein characterization		
Dynamic light scattering (DLS)	Soluble protein	—
Mass spectrometry	Soluble protein	—
Crystallography		
Crystal screen	Soluble protein	Crystal
Crystal optimization	Soluble protein	Crystal
Crystal harvest	Crystal	Mounted crystal
Test diffraction	Mounted crystal	—
Diffraction	Mounted crystal	—
